# A highly pH-sensitive nanowire field-effect transistor based on silicon on insulator

**DOI:** 10.3762/bjnano.4.38

**Published:** 2013-05-28

**Authors:** Denis E Presnov, Sergey V Amitonov, Pavel A Krutitskii, Valentina V Kolybasova, Igor A Devyatov, Vladimir A Krupenin, Igor I Soloviev

**Affiliations:** 1 Lomonosov Moscow State University Skobeltsyn Institute of Nuclear Physics, Moscow 119991, Russia; 2 Laboratory of Cryoelectronics, Lomonosov Moscow State University, Moscow 119991, Russia; 3 Keldysh Institute of Applied Mathematics, Moscow 125047, Russia

**Keywords:** charge/field sensor, field-effect transistor, nanowire, pH sensor, silicon-on-insulator

## Abstract

**Background:** An experimental and theoretical study of a silicon-nanowire field-effect transistor made of silicon on insulator by CMOS-compatible methods is presented.

**Results:** A maximum Nernstian sensitivity to pH change of 59 mV/pH was obtained experimentally. The maximum charge sensitivity of the sensor was estimated to be on the order of a thousandth of the electron charge in subthreshold mode.

**Conclusion:** The sensitivity obtained for our sensor built in the CMOS-compatible top-down approach does not yield to the one of sensors built in bottom-up approaches. This provides a good background for the development of CMOS-compatible probes with primary signal processing on-chip.

## Introduction

Over the past decade experimental and theoretical studies of semiconductor nanowire field-effect transistors (NW FET) made of silicon on insulator (SOI) have been of great interest to researchers. The large surface-to-volume ratio of the nanowire allows one to create extremely sensitive charge/field sensors in chemical and biological systems for the detection of charged particles and molecules at low concentrations [1–4]. It was shown [5] that the charge sensitivity of NW FET can reach a value of 60 · 10^−6^*e*/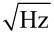 at 198 K (*e* is the electron charge), which is orders of magnitude better than conventional FET and nanomechanical systems. This extremely high sensitivity gives an opportunity to construct local potential probes with nanoscale lateral resolution based on NW FET. In comparison with single-electron transistors [6], it is easy to fabricate a device operating at room temperature, which can be useful for biological and medical applications. A demonstration of such a local probe based on a vapour–liquid–solid-method (VLS) grown silicon-nanowire (SiNW) FET was given in [7]. The sensitivity of this bioprobe to pH change near its maximum value of 59 mV per unit pH was reached and the intracellular electrical recording from beating cardiomyocytes was demonstrated. It was shown that this sensor charge sensitivity in subthreshold mode was around several tens of *e*. These outstanding results were obtained by methods incompatible with traditional semiconductor electronics.

In this work we present SiNW FET fabricated [8] by traditional methods from silicon-on-insulator (SOI) with a pH sensitivity equal to VLS-grown NW FET [7]. The maximum sensitivity in subthreshold mode is estimated to be on the order of 10^−3^*e*/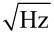.

## Results and Discussion

**Figure 1:**
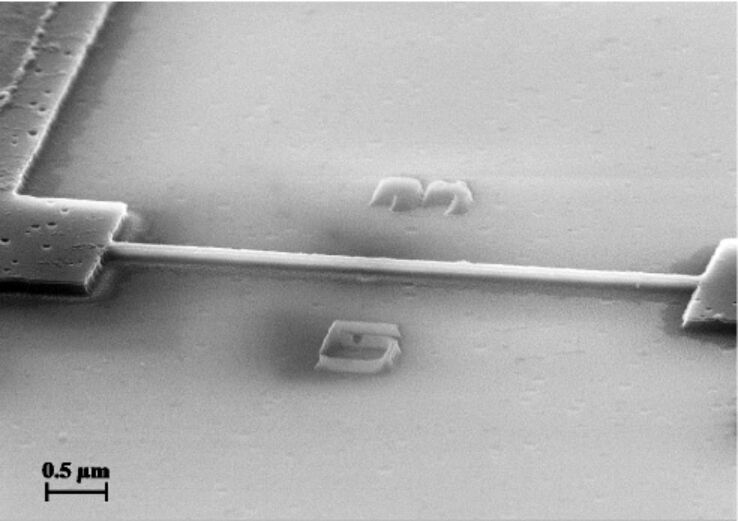
SEM image of the nanowire and the contact pads. The length of the nanowire is 5 μm, the width is 100 nm.

In Figure 1 a NW FET with a channel length of 5 μm and a width of 100 nm is presented. We used Soitec SOI wafers with a device layer of 55 nm and a buried oxide layer of 145 nm. The device layer is boron doped with a concentration of about 10^15^ cm^−3^. The fabrication steps included [9]

1. Electron-beam lithography in positive resist to pattern the image of the NW and contact pads.
2. Aluminium mask e-beam vapour deposition.
3. Anisotropic reactive ion etching of the device layer through the Al mask and mask removal.
4. Magnetron sputtering of titanium electrodes and their isolation with silica to allow measurements in liquids.

Both optical and electron-beam lithography was used to pattern electrodes and for isolation. The thickness of the Ti and SiO_2_ layers was 50 and 200 nm, respectively. Schottky barriers are formed between silicon contact pads and Ti electrodes. Fabricated transistors were studied in air and in buffer solutions with different pH values. The sensitivity of a semiconductor sensor strongly depends on the charge carrier density, which can be changed by the gate voltage *V*_g_ applied to the SOI handle layer. During pH measurements, the liquid itself serves as a second gate with a voltage *V*_ref_. The measured transistor characteristics were strongly asymmetric. The hole conductivity of the transistor was very low down to gate voltages of *V*_g_ = −10 V. For positive voltages at the gate (when an inverse electron channel formed), typical *I*–*V*-curves with ohmic and saturation regions were measured. Such characteristic asymmetry is induced by a Schottky barrier. Its height is different for electrons and holes [10]. For p-type silicon (p-Si) with a doping level of about 10^15^ cm^−3^ and Ti electrodes, the barrier height for holes is about three times higher than for electrons. Measurements of the pH sensitivity of the transistor were carried out at positive voltages *V*_g_ at the gate. The measurements were carried out by applying fixed source–drain and source–gate voltages *V*_sd_ = −0.5 V and *V*_g_ = 8 V, respectively, and measuring the resulting transport current. The inversion channel that forms under these conditions in p-Si is optimal for pH measurements in liquids [11]. An AgCl electrode dipped in a buffer solution was used as a reference electrode. The pH measurements were carried out statically in droplets without a flux of liquid. Large amounts of buffer solution with the target pH were pumped through the droplet volume to change the pH level.

**Figure 2:**
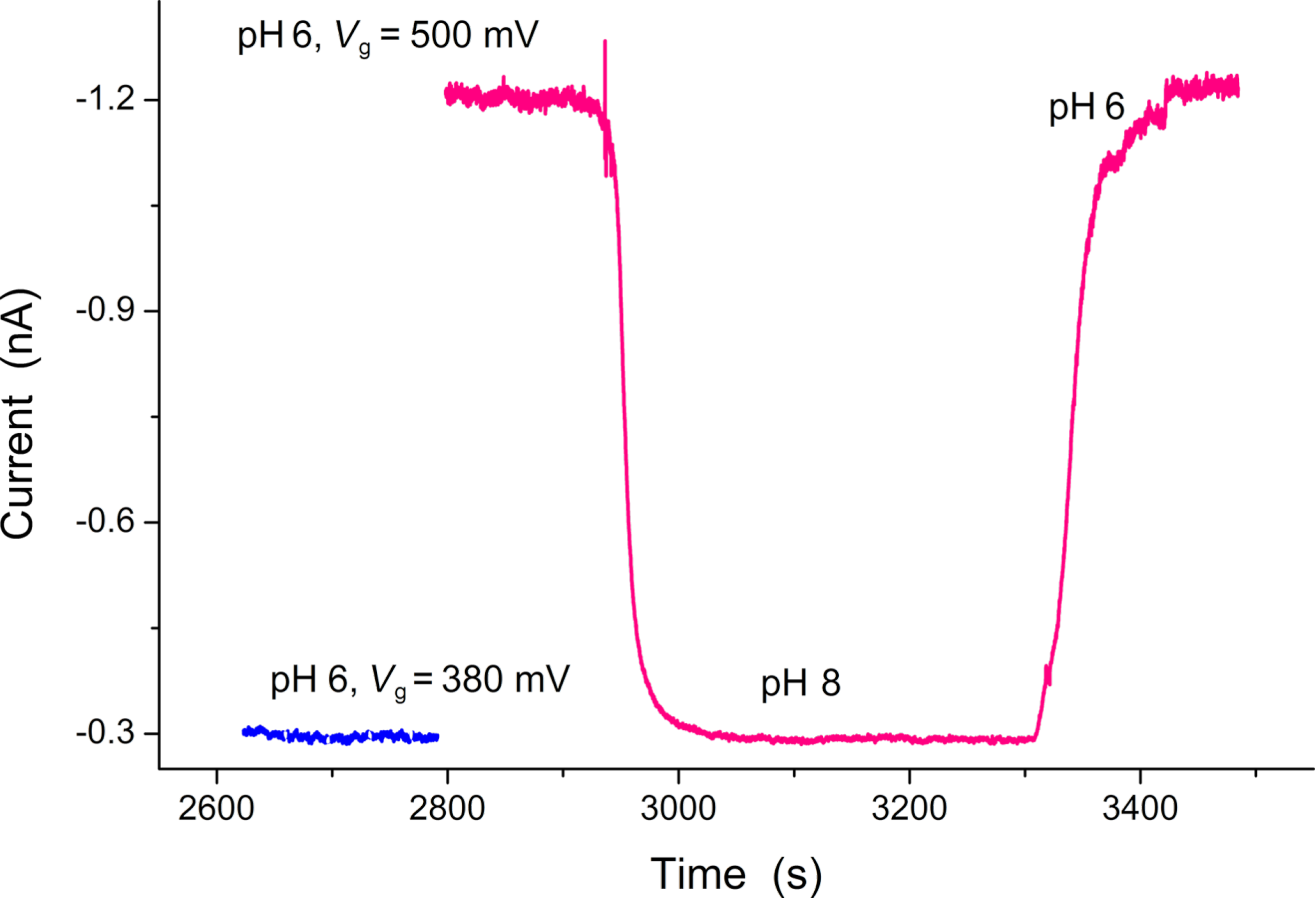
SiNW FET response to the change of the pH value of the buffer solution.

In Figure 2 the transport current of the SiNW FET at different pH values of the buffer solution and at different reference-electrode potentials is shown.

One can see that the current level for a transistor in buffer solution at pH 8 and *V*_ref_ = 0.5 V coincides with the current for a buffer solution at pH 6 and *V*_ref_ = 0.38 V. Accordingly, the pH sensitivity of the SiNW FET (i.e., the change of the insulator–electrolyte potential, Ψ_0_, to a change of the bulk pH [12]) can be estimated as:

[1]
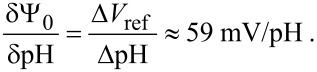


This is an extremely high value for an ion-sensitive FET (ISFET) with silica as a gate dielectric. It is comparable with the sensitivity of ISFETs with special gate dielectrics such as Ta_2_O_5_. Moreover this sensitivity is comparable to the theoretical limitation at room temperature [12].

Field/charge sensors are traditionally characterized by a maximum charge sensitivity. To estimate this, we measured the NW FET conductivity dependence on the charge at the surface of the NW native oxide layer and calculated the spectral density of transport current fluctuations. We used linearised Poisson–Boltzmann equations to define the electrical potential in the NW and in the electrolyte together with the Poisson equation for the electrical potential in the NW oxide layer. The exact solution of this three-layer problem, as opposed to the estimations of Gao et al. [13], allows us to explore the potential profile explicitly. A linearisation of the Poisson–Boltzmann equations is possible in the case of weak potentials applied to the reference electrode and a small bending of semiconductor bands, so that |*e*φ_s/ox_|,|*e*φ_ox/el_| < *k*_B_*T*, where φ_s/ox_ and *φ*_ox/el_ are the potentials at the nanowire–oxide and oxide–electrolyte boundaries, respectively, *k*_B_ is the Boltzmann constant and *T* is the temperature. Numerical methods used by several authors previously [11,14–16] for solving the Poisson–Boltzmann equation do not allows one to clearly demonstrate the behaviour of the studied system in different modes.

In our calculations we assumed the absence of charges inside the oxide layer and a uniformity of the dopant density in the NW as in [13]. Moreover, we assumed that the electric field, which is directed normally to the NW surface, is much larger than the longitudinal one so that the latter does not influence the modulation of the NW conductivity [11]. This assumption is correct because the NW length is much larger than its lateral dimensions and the voltage at the contacts is low. As in previous reports [11,13–14], we assumed that electrolyte ions can come directly to the oxide layer surface. Using a cylindrical coordinate system ***r*** = ***r***(ρ, α, *z*) with the *z* axis directed along the NW axis, the equations become [17]

[2]
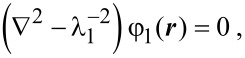


[3]
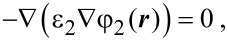


[4]
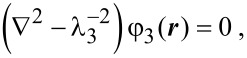


where φ_1_, φ_2_, and φ_3_ are the potentials in the nanowire, oxide layer, and electrolyte, respectively (regions 1, 2, 3). The parameters λ_1_ and λ_3_ are the Debye lengths of screening in the NW and electrolyte, respectively. The boundary conditions of the problem are the equality of the potentials and electric displacement fields at the nanowire–oxide interface and the equality of the leap in potentials and electric-displacement fields due to charges at the oxide–electrolyte interface. The potential at infinity approaches zero. Taking into account the axial symmetry of the nanowire, we obtain as the solution for the potential inside the nanowire

[5]
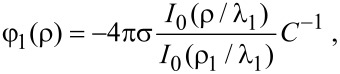


where σ is the surface charge density at the oxide–electrolyte interface, ρ_1_ is the nanowire radius, *I*_0_ the modified Bessel function of the first kind to zeroth order, and *C* is the off-diagonal coefficient of the capacitance matrix [18] that is responsible for the change in potential at the nanowire–oxide interface due to the variation in surface charge density at the oxide–electrolyte interface. This capacitance is defined by geometrical and electrical parameters of the system:

[6]
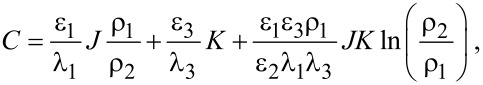


where ε_1_, ε_2_, and ε_3_ are the relative permittivities in regions 1, 2, and 3, respectively, ρ*_2_* = ρ_1_ + δρ with δρ being the oxide-layer thickness, and


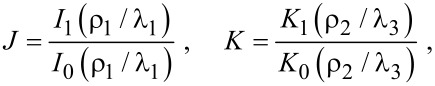


where *I*_0,1_ and *K*_0,1_ are modified Bessel functions of the first and second kind, respectively.

In Equation 6, the two first terms are responsible for the capacitance of the NW and electrolyte. It is seen that for ρ_1_ ≈ ρ_2_ the last term in Equation 6 becomes a product of the two first term multiplied by ≈δρ/ε_2_ and is responsible for the oxide layer. With real coefficient values substituted in Equation 6, the second term becomes 10 times greater than the first one. The response of the NW FET to the variation of surface charge can be found by inserting Equation 5 in the equation for the relative NW conductivity modulation [17]

[7]
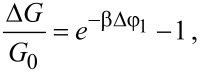


where β = *e*/*k*_B_*T*. The linearisation of Equation 7 by Δφ_1_ (such that 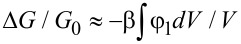, where *V* is the NW volume) allows one to obtain a simple expression for the response of the transistor in the linear mode:

[8]
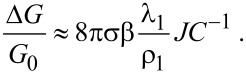


Under transition to the subthreshold mode, the concentration of charge carriers in the NW decreases by orders of magnitude and the screening length in the NW, λ_1_, increases accordingly. Simultaneously, the dependence of the potential on the coordinate in Equation 5 disappears (*I*_0_(*x*) ≈ 1, *x* → 0), the NW capacitance decreases and only the second term remains in Equation 6 (λ_1_ >> ρ_1_, *J* → ρ_1_/2λ_1_) so that the potential in the NW does not depend on the electrical parameters of the NW and the oxide layer:

[9]
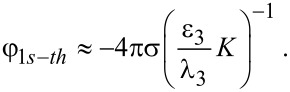


The product in the brackets of Equation 9, which corresponds to Equation 6, is the self-capacitance of the studied system in the subthreshold mode. This capacitance coincides with the capacitance of the electrolyte double-layer. This result corresponds to the expression for the full capacitance of the system from [13] for the considered mode. To estimate the NW FET response in subthreshold mode, one should substitute Equation 9 into Equation 7. It should be noted that the response of the transistor to variation of the surface charge density in this mode is exponential [17]. Considering the estimation for the mobility of charge carriers in the inversion channel from [10] for our measurements (Figure 2) we get λ_1_ >> ρ*_1_*, so our approximation in Equation 9 is appropriate in this case. Using Equation 9, we get an estimation of the charge variation at the NW surface Δ*Q* ≈ 5 × 10^4^ *e* for a change of the pH value from 8 to 6. This value is one order of magnitude larger than the one in the report of Gao et al. [13]. The difference can be explained by the NW surface area. In our case the NW radius was ≈100 nm, while in Gao’s case [13] it was only 5 nm. Moreover, the pH sensitivity of our sample is two times higher.

In the case of the application of NW FET to biosensors it is necessary to consider the large dimensions of the molecules. The detected charge will be located not on the surface but in the electrolyte double-layer. This region can be modeled [19] as an ion-permeable membrane with the accordingly changed Poisson–Boltzmann equation for it. While the exact solution of this problem can be found by numerical methods, one often resorts to a simplified model [20], which qualitatively correctly describes the system under study. To take into account the dipole moment of the detected molecules one should reformulate the boundary conditions [14] by adding the leap of the potential at the oxide–electrolyte interface. This will lead to the following correction of Equation 6:

[10]
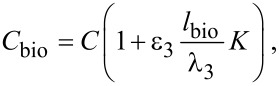


where *l*_bio_ is the effective thickness of the layer, and the dipole moment can be represented by τ_bio_ = σ*l*_bio_.

The charge sensitivity of the NW FET is

[11]
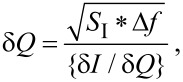


here *S*_I_ is the spectral density of the NW current fluctuations, Δ*f* is the output frequency band of the device and is assumed to be 1 Hz, δ*I*/δ*Q* is the NW current response on the surface–charge variation. In general, the spectral density of current fluctuations *S*_I_ is determined by noncoherent contributions of the substrate and electrolyte noise and intrinsic current fluctuations of the NW FET [21]. It was shown [22] experimentally that the fluctuation of electrolyte ions can be neglected. The substrate noise is 1/*f* noise and it is important to take it into account in a direct low-frequency readout from the NW FET [21–22]. However, the 1/*f*-noise intensity rapidly decreases with the readout frequency increasing and it plateaus out at *f* ≈ 2 kHz in [5] or *f* ≈ 80 Hz in [21]. Therefore the lock-in technique [5] and the correlation analysis (simultaneous measurement by several equal devices) that we used allow us to eliminate 1/*f*-noise. Thus, the maximum NW FET sensitivity is defined by the frequency-independent component of the spectral density *S*_I_.

The spectral density of NW current fluctuations at an angular frequency ω = 2π*f*, so that 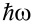 << *k*_B_*T*, is determined by the thermal fluctuation *S*_In_ = 4*k*_B_*TG*. The spectral density of current fluctuations at Schottky barriers formed at contact regions is described by [23] as *S*_IB_ = (2*eV*/*R*_B_) coth (*eV*/2*k*_B_*T*_B_), where *T*_B_ is the temperature of the barrier, *R*_B_ is the barrier resistance, and *V* the voltage drop across it. At *eV* << *k*_B_*T*_B_ this equation turns into the thermal fluctuation equation *S*_IB_ = 4*k*_B_*T*/*R*_B_; at *eV* >> *k*_B_*T*, it turns into shot noise equation *S*_IB_ = 2*eI*. Since the distance between the NW and the Schottky barriers in our design is about several microns, which is far larger than the phase-breaking length [24], current fluctuations in the NW and in the Schottky barrier were uncorrelated. According to this, one can calculate the spectral density of the transport-current fluctuations *S*_I_ by considering an equivalent scheme with a series connection of resistors modelling NW and Schottky barriers with uncorrelated fluctuation generators:

[12]
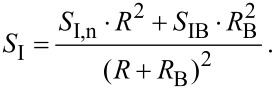


Our four-probe measurements of the NW resistance *R* and Schottky barrier resistance *R*_B_ at room temperature show that *R* >> *R*_B_ ≈ 1 kΩ (applied voltage *V*_d_ = 0.5 V). Rough estimates at the values of the parameters show that the spectral density of the current fluctuations at the Schottky barriers is described by the thermal-fluctuation equation and its contribution to *S*_I_ in Equation 12 is negligibly small in comparison with the NW current fluctuations. Considering this, from Equation 11 and Equation 12 it follows that the charge sensitivity of NW FET is

[13]
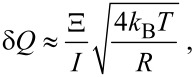


where Ξ = ρ_2_*LK*ε_3_/2βλ_3_, *L* is the NW length, and *I* the direct current through the barriers. As opposed to the respective expression in [5] there is no shot-noise contribution proportional to the current in our estimation of the fluctuation. The derived Equation 13 is more correct since it is known [24] that in diffused resistors shot noise does not sum up to the thermal fluctuation and exists only for resistors of small size at low temperature. Equation 13 gives an estimation of the maximum NW FET charge-sensitivity in subthreshold mode δ*Q* ≈ 5 × 10^−3^*e*/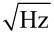 for our measurements. This derived value is better than the results obtained in [13], which can be explained by a better pH sensitivity of our transistor.

## Conclusion

In this work we demonstrated experimentally the possibility of the fabrication of a highly sensitive pH sensor and charge sensor based on NW FET made from SOI using traditional semiconductor technology. The conducted analysis of the model allows us to estimate the value of the NW relative-conductivity modulation due to the variation of the charge density on the oxide–electrolyte interface as well as the variation of this charge density due to the pH variation for a known pH sensitivity of the NW FET. The calculated maximum charge sensitivity in subthreshold mode is estimated to be 5 × 10^−3^*e*/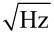. The pH sensitivity of our experimental samples is close to the theoretical limit of 59 mV/pH and is not inferior to VLS-grown nanowires [7,13]. It was shown that the simplified fabrication technology with Schottky barriers in contact regions allows one to avoid processes of doping and dopant activation and has no effect on the NW transport-current fluctuation density.
